# Differential Regulation of Disheveled in a Novel Vegetal Cortical Domain in Sea Urchin Eggs and Embryos: Implications for the Localized Activation of Canonical Wnt Signaling

**DOI:** 10.1371/journal.pone.0080693

**Published:** 2013-11-13

**Authors:** ChiehFu Jeff Peng, Athula H. Wikramanayake

**Affiliations:** Department of Biology, University of Miami, Coral Gables, Florida, United States of America; University of North Carolina at Chapel Hill, United States of America

## Abstract

Pattern formation along the animal-vegetal (AV) axis in sea urchin embryos is initiated when canonical Wnt (cWnt) signaling is activated in vegetal blastomeres. The mechanisms that restrict cWnt signaling to vegetal blastomeres are not well understood, but there is increasing evidence that the egg’s vegetal cortex plays a critical role in this process by mediating localized “activation” of Disheveled (Dsh). To investigate how Dsh activity is regulated along the AV axis, sea urchin-specific Dsh antibodies were used to examine expression, subcellular localization, and post-translational modification of Dsh during development. Dsh is broadly expressed during early sea urchin development, but immunolocalization studies revealed that this protein is enriched in a punctate pattern in a novel vegetal cortical domain (VCD) in the egg. Vegetal blastomeres inherit this VCD during embryogenesis, and at the 60-cell stage Dsh puncta are seen in all cells that display nuclear β-catenin. Analysis of Dsh post-translational modification using two-dimensional Western blot analysis revealed that compared to Dsh pools in the bulk cytoplasm, this protein is differentially modified in the VCD and in the 16-cell stage micromeres that partially inherit this domain. Dsh localization to the VCD is not directly affected by disruption of microfilaments and microtubules, but unexpectedly, microfilament disruption led to degradation of all the Dsh pools in unfertilized eggs over a period of incubation suggesting that microfilament integrity is required for maintaining Dsh stability. These results demonstrate that a pool of differentially modified Dsh in the VCD is selectively inherited by the vegetal blastomeres that activate cWnt signaling in early embryos, and suggests that this domain functions as a scaffold for localized Dsh activation. Localized cWnt activation regulates AV axis patterning in many metazoan embryos. Hence, it is possible that the VCD is an evolutionarily conserved cytoarchitectural domain that specifies the AV axis in metazoan ova.

## Introduction

 How animal body plans are established during embryogenesis is a central question in developmental biology. Animals are morphologically diverse, but in bilaterians, which comprise the vast majority of metazoan taxa, the body plans are built around three distinct embryonic coordinates that define the anterior-posterior (AP), the dorsal-ventral (DV) and the left-right (LR) axes. In most bilaterian taxa the AP axis is considered to be the primary embryonic polarity, and its provenance is strongly linked to the animal-vegetal (AV) axis, a primordial polarity present in the unfertilized ovum [1,2]. The relationship between the AV egg polarity and the AP axis was first reported in the early 19^th^ century by Karl Ernst von Baer [3]. In this work Baer reported that one pole of the amphibian egg corresponded to the future anterior end of the embryo, and blastomeres derived from this pole gave rise to the epidermis, the central nervous system, and the sense organs, while the cells derived from the opposite pole gave rise to the endoderm, the internal organs, and the posterior end of the embryo. Many studies have since confirmed that the eggs of most metazoans have an AV axis, and moreover, that the relationship between this egg polarity and patterning of the AP axis first observed in amphibians by Baer is conserved in many other bilaterians [1,2,4-10]. Moreover, recent studies in a number of model organisms have established a role for the evolutionarily conserved canonical Wnt (cWnt) signaling in regulating pattern formation along the AP axis. But how the asymmetric activation of this pathway is influenced by the AV axis of the egg is still not well understood in most metazoans and as such it is an important area of investigation [5,6,11,12]. 

 The sea urchin embryo has proven to be a useful system for studying early specification events that initiate pattern formation along the AV axis. Early cell fate specification in this embryo follows the canonical bilaterian pattern with the animal half blastomeres giving rise to epidermal and neural cell types in the pluteus larva, and the vegetal blastomeres becoming specified as endomesoderm very early in embryogenesis [13-15]. A pivotal early event occurs at the fourth cleavage when the asymmetric division of the four vegetal blastomeres of 8-cell stage embryos gives rise to four micromeres at the vegetal pole. Signals produced by these 16-cell stage micromeres induce endomesoderm specification in overlying macromeres thereby initiating patterning along the AV axis [13]. Experimental studies have implicated the presence of localized maternal determinants for endomesoderm specification in sea urchins but the identity of these factors has remained elusive. Molecular studies have shown however, that an early function for these localized maternal determinants is to activate the cWnt pathway in vegetal blastomeres [16-18]. The cWnt pathway is normally activated when a Wnt ligand binds to the LRP5/6 and Frizzled (Fz) receptor complex, and “activates” the cytoplasmic phosphoprotein Disheveled (Dsh) [19,20]. How the receptor complex regulates Dsh activity is not well understood, but an immediate downstream consequence is that activated Dsh disrupts the targeted degradation of the cytoplasmic protein β-catenin by a destruction complex that primarily contains the proteins APC (product of the *adenomatous polyposis coli* gene), glycogen synthase kinase-3β (GSK-3β) and Axin. Disruption of the destruction complex-mediated degradation of β-catenin leads to this protein accumulating in the cytoplasm and subsequently translocating into the nucleus where it heterodimerizes with DNA-binding proteins such as Lef/Tcf and functions as a transcriptional coactivator of target genes [19,20]. Hence, nuclearization of β-catenin is in general a reliable qualitative measure of activation of cWnt signaling.

 There is now a body of data that provides compelling evidence that localized activation of cWnt signaling in vegetal blastomeres is critical for early pattern formation in the sea urchin embryo [17,18,21-23]. The β-catenin protein is initially nuclearized in the micromeres of 16-cell stage embryos, and at the 32-cell stage the protein is also detected in nuclei of the overlying macromere cell tier [17]. During the transition from the 32- to 60-cell stage, the macromeres divide equatorially to produce two cell tiers, with the tier closer to the animal pole referred to as the veg1 and the tier that forms closer to the vegetal pole referred to as veg2. Nuclear β-catenin is downregulated in the veg1 tier, but the protein remains at high levels in the nuclei of the veg2 tier and the micromeres. Hence, at the 60-cell stage nuclear β-catenin marks all cells that are specified as endomesoderm at this stage [17,24]. Consistent with the nuclear localization of β-catenin in vegetal cells, functional molecular studies have established that activation of cWnt signaling is the critical input for endomesoderm specification and pattern formation along the AV axis [16-18]. Direct downstream targets of β-catenin include a set of signaling molecules and transcription factors that activate the now well-described endomesodermal gene regulatory network (EGRN) [25,26]. Continuing work has defined many of the sub-circuits mediating various specification events during early embryogenesis in the sea urchin, and the sea urchin EGRN currently is one of the best described GRNs regulating early pattern formation in embryos [13,26,27]. 

 In contrast to the extensive current knowledge on the regulation and interaction of GRN components downstream of β-catenin, the mechanisms that selectively activate nuclear β-catenin signaling in vegetal cells are not as well understood. The nuclearization of β-catenin is usually regulated by a ligand-receptor interaction at the cell surface. Hence, one logical mechanism for the selective activation of cWnt signaling in the early sea urchin embryo is that there is a maternally expressed Wnt ligand or receptor localized to the vegetal pole. In sea urchins it is known that four of the eleven Wnts encoded in the *Strongylocentrotus purpuratus* genome (*Wnt6*, *Wnt7*, *Wnt16*, and *WntA*) are maternally expressed [21,28-30]. However, although maternal Wnt6 has been shown to be required for activation of the Wnt pathway in macromeres and their descendants, neither *Wnt6* nor any of the other maternally expressed Wnt ligands display localized expression in the egg or early embryo [21,28,30]. Similarly, while Fz1/2/7 has been shown to be required for activation of the cWnt pathway in the macromere lineage (in *Paracentrotus lividus*), neither this Fz receptor nor any of the other known Wnt receptors encoded in the sea urchin genome (*SpFz4*, *SpFz7*, *SpFz5/8*, *SpFz9/10*, and *SpLrp6*) that are maternally expressed display a localized expression pattern at the vegetal pole at the mRNA level [21,22,28,30]. Hence, while specific cell surface components are required for activation of cWnt signaling in the early sea urchin embryo, these factors do not appear to mediate the specificity of localized activation of cWnt signaling in vegetal blastomeres. 

 Recent work has shown that Dsh, a central upstream component of the Wnt signaling pathway, plays a critical role in mediating the localized activation of the EGRN in vegetal cells in the early sea urchin embryo [31]. The results from these studies have raised the possibility that the Dsh protein is locally “activated” in vegetal cells, and moreover, that this activation step requires the vegetal cortex of the egg [21,31]. *Dsh* is maternally expressed and like the other maternally expressed components of the cWnt pathway *Dsh* mRNA expression is uniform in the egg and during early embryogenesis [30,31]. However, when a Dsh::GFP fusion protein was overexpressed by mRNA injection into fertilized eggs, accumulation of Dsh::GFP was seen at the vegetal cortex as early as the zygote stage, and endogenous Dsh protein was shown to be enriched at the vegetal pole of unfertilized eggs in the sea urchin *P. lividus* [21,31,32]. Interfering with Dsh function in the cWnt pathway using a dominant-negative form of Dsh blocked nuclearization of endogenous β-catenin and activation of the EGRN [31]. Strikingly however, overexpression of Dsh by mRNA injection into zygotes had little visible effect of embryonic development [31]. This result was unexpected since overexpression of constitutively active forms of β-catenin and Lef/Tcf, or a dominant-negative GSK-3β severely vegetalizes embryos by respecifying animal half blastomeres as endomesoderm [16,18,33]. The accumulation of Dsh at the vegetal cortex and the failure of overexpressed Dsh to activate cWnt signaling in animal half blastomeres raised the possibility that the vegetal egg cortex is required for Dsh “activation” during endomesoderm specification. Support for this idea has come from recent embryological experiments where unfertilized eggs were stripped of the vegetal egg cortex and then fertilized and allowed to develop [21]. Embryos developing from these vegetal cortex-deleted eggs did not form endomesoderm and became animalized. These embryos could be completely rescued by injection of *β-catenin* mRNA, or partially rescued by injection of *Dsh* mRNA. Moreover, when an extirpated vegetal cortex was transplanted and fused to the animal pole of a host egg, ectopic endoderm was induced suggesting that crucial maternal factors that activate cWnt signaling are localized at the vegetal cortical region [21]. These and other observations previously discussed underscore the importance of a pool of Dsh closely associated with the vegetal cortex in activation of Dsh during cWnt signaling in the early sea urchin embryo. However, little is known about the structure of the vegetal cortex region that binds Dsh::GFP, and when and how endogenous Dsh begins to be localized to this domain. Additionally, nothing is known about how the Dsh protein is differentially regulated in vegetal blastomeres during cWnt signaling. Elucidation of these questions is crucial for a comprehensive understanding how the sea urchin AV axis is initially specified.

 In the current study we use affinity-purified polyclonal antibodies to examine the localization and differential modification of the endogenous Dsh protein in the sea urchin egg and early embryo. We show that while Dsh is widely expressed in the egg and during early development, a differentially modified form of Dsh accumulates in a novel domain at the vegetal egg cortex that we have termed the vegetal cortical domain (VCD). During early cleavage stages the VCD and the associated Dsh puncta are inherited by all vegetal cells that activate cWnt signaling in the early embryo. We also show that Dsh accumulation at the VCD begins during early oogenesis and it strikingly correlates with the appearance of the centrosome at the animal pole. Experiments to determine if the cytoskeleton plays a role in the tethering of Dsh to the VCD revealed that microfilaments and microtubules are not directly involved in tethering this protein to the VCD. Unexpectedly, however, disruption of microfilaments led to the degradation of all Dsh pools in the unfertilized egg over a period of incubation indicating that microfilament integrity is required for maintenance of Dsh stability in the egg. These results provide novel insights into the origins of AV axis polarity in the sea urchin egg, and moreover, provide insight into the mechanisms that lead to the selective activation of cWnt signaling in vegetal cells during early cleavage stages in the sea urchin embryo.

## Results

### Dsh is broadly expressed during early embryogenesis, but the protein is highly enriched at the vegetal cortex

**Figure 1:**
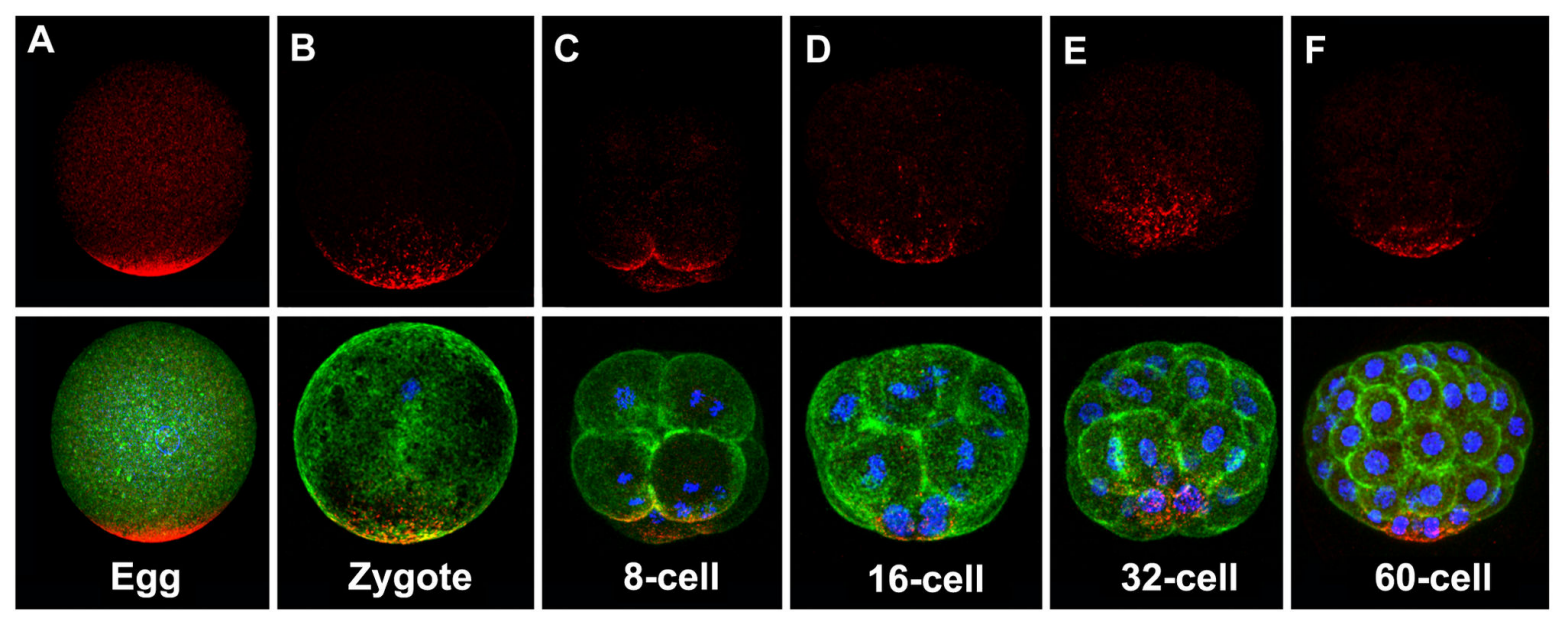
Disheveled protein is highly enriched at the vegetal pole of eggs and early embryos. *S*. *purpuratus* eggs and embryos were processed for immunofluorescence and analyzed using scanning confocal microscopy. (A-F) Developmental stages from an unfertilized egg to a 60-cell stage embryo. Dsh was immunolocalized using an anti-Dsh antibody (red), filamentous actin was visualized using fluorescein phalloidin (green), and nuclei were visualized using DAPI (blue). Top panels show Dsh staining and the corresponding bottom panels show an overlay of Dsh with phalloidin and DAPI. All images are oriented with the animal pole towards the top and vegetal pole towards the bottom. (A) An unfertilized egg showing the asymmetric enrichment of Dsh at one pole. (B) Zygote stage. After fertilization the Dsh pattern becomes more punctate. (C) 8-cell stage embryo. Punctate Dsh staining is seen in the four cells. (D) 16-cell stage embryo. Punctate Dsh staining is observed in the micromeres and the macromeres indicating that the Dsh accumulation at earlier stages is at the vegetal pole. (E) 32-cell stage embryo. Punctate Dsh staining is seen in the macromeres and the vegetal tier cells. (F) 60-cell stage embryo. Dsh puncta are seen predominantly in the micromeres and the veg2 tier.

 Previous studies have shown that *Dsh* transcripts are expressed uniformly in eggs and early embryos and this observation was confirmed here [31] (Figure S1A). To examine the expression of the Dsh protein we performed Western blot analysis on lysates collected from eggs and several early developmental stages of sea urchin embryos using the anti-SUDshDIX polyclonal antibodies. These experiments showed that congruent with the expression pattern of *Dsh* transcripts the protein is expressed maternally and throughout embryogenesis (Figure S1B). These polyclonal antibodies recognized a major band at approximately 85 kD, which is larger than the predicted size for the SpDsh protein at 81 kD suggesting that the protein is post-translationally modified. To validate the specificity of the anti-SUDshDIX antibodies we carried out preadsorbtion experiments and the results of these studies supported the specificity of these polyclonal antibodies for Western blot analysis (Figure S2A). We next used the polyclonal antibodies to determine the spatial distribution of the endogenous Dsh protein in eggs and early embryos. Staining of unfertilized eggs revealed a striking asymmetric enrichment of Dsh at the cell cortex (Figure 1A; Figure S2B) confirming earlier observations in *P. lividus* [21]. Immunolocalization of Dsh following fertilization and during the early cleavage stages showed that the enrichment of this protein on one side persisted through the late cleavage stages, and expression in the micromeres and the macromeres at the 16-cell stage confirmed that the early cortical expression was at the vegetal pole (Figure 1B-D). By the 32- and 60-cell stages, the cortically-enriched domain of Dsh correlated well with the previously reported domain of nuclear β-catenin in vegetal blastomeres (Figure 1E, F) [17]. This staining pattern was obtained with all three anti-SUDsh antibodies. 

 In unfertilized eggs (Figure 1A) and during the cleavage stages (Figure 1C-F), it appeared that there was diffuse cytoplasmic Dsh staining in the animal half of eggs and embryos. To confirm that the Dsh protein is present in animal pole blastomeres Western blot analysis was carried out on isolated mesomeres and macromere/micromere pairs collected from 16-cell stage embryos. This analysis showed that the Dsh protein is clearly present in animal pole blastomeres (Figure S1C). We conclude that the Dsh protein has a broad distribution in the unfertilized egg and early embryos, but that the protein is enriched at the vegetal pole cortex. 

### A pool of Dsh protein is embedded in the egg cortex

**Figure 2:**
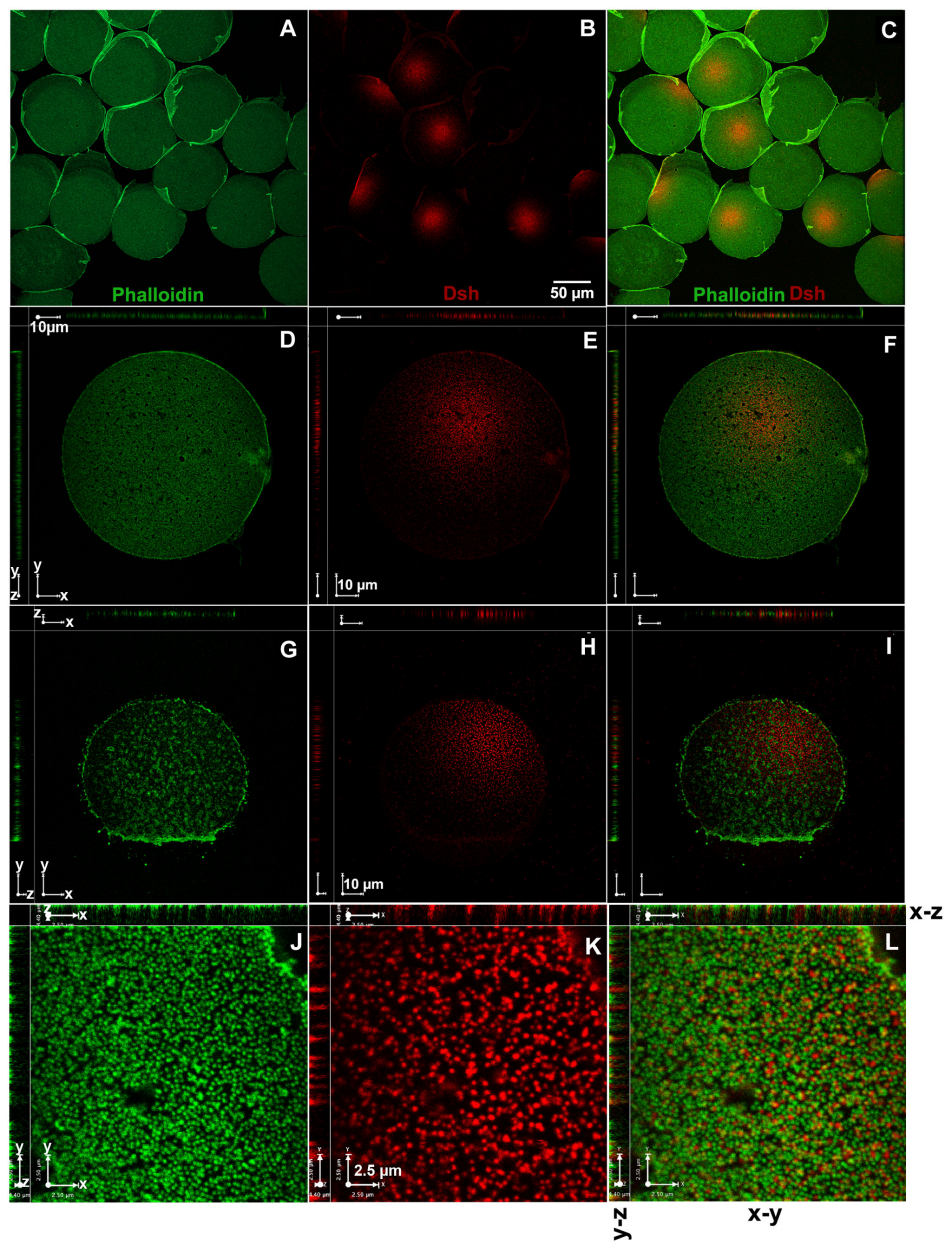
The Disheveled protein enriched at the vegetal pole is embedded in a vegetal cortical domain. Cortices were collected from S. purpuratus eggs and zygotes, prepared for anti-Dsh immunofluorescence and viewed using scanning confocal microscopy. (A-C) Wide field view of cortices isolated from unfertilized eggs shows the accumulation of Dsh in the vegetal cortical domain (—, 50µm). (D-F) A high magnification view of a single cortex labeled by anti-Dsh antibodies and phalloidin shows a uniform F-actin distribution and concentrated Dsh in one domain, (—, 10µm). Note the punctate appearance of the Dsh staining. (G-I) A cortex isolated from a zygote labeled with anti-Dsh antibodies and phalloidin shows that Dsh remains anchored in the vegetal cortical domain following fertilization (—, 10µm). (J-L) A higher magnification view of the Dsh puncta at the vegetal cortex shows that Dsh is embedded between short actin filaments, (—, 2.5µm). Insets on the left and top of each panel from D-L shows a 90˚ rotation of the 3D confocal dataset shown in D-L (x-y) and allows the same image to be viewed in cross sections, x-z and z-y, showing Dsh is embedded between short actin filaments.

 Immunostaining of eggs and early embryos showed a striking vegetal enrichment of Dsh suggesting that the protein was accumulating in a domain closely associated with the vegetal cortex. This region has previously been described as the vegetal cortical domain (VCD) [41]. To further investigate the association between Dsh and the VCD we immunolocalized the protein in isolated egg cortices. Cortical lawns were collected and processed for immunofluorescence using the anti-SUDsh antibodies. Analysis of these samples using fluorescence and scanning confocal microscopy showed that there is a striking localization of the Dsh protein in the isolated cortical fragments (Figure 2A-F). Immunostaining of cortices isolated from zygotes also showed the localized domain of Dsh indicating that the VCD is retained after fertilization (Figure 2G-I). While we cannot isolate cell cortices from later blastomeres the continued accumulation of Dsh in the cortex of vegetal cells indicates that the VCD is inherited by the micromeres and the macromeres and retained in these cells at least through the 60-cell stage of development (Figure 1). To the best of our knowledge, the VCD, as defined by Dsh accumulation, is the first cytoarchitectural asymmetry reported to be present in the sea urchin egg cortex at the vegetal pole.

 Dsh immunolocalization in isolated cortices also revealed some additional features of the VCD that were notable. In the immunolocalization experiments, the cortices were also stained with fluorescein phalloidin to localize filamentous actin (F-actin). Superimposing the Dsh localization pattern with F-actin staining revealed that in many cases, the Dsh puncta appeared to be embedded between actin microfilaments and these were most likely associated with short microvilli (Figure 2J-L). However, there was only a slight overlap between the Dsh puncta and the actin microfilaments. We also noted that the VCD is a circular domain and that there appears to be a concentration gradient of Dsh puncta with the highest concentration of these puncta in the center of the VCD, which decreased as the domain extended towards the animal pole (Figure 2D-I). These observations raise the possibility that the egg cortex may embed polarity information that is used during early development of the sea urchin embryo.

### Dsh protein bound to the vegetal cortical domain is differentially modified compared to the isoforms found in the bulk cytoplasm

**Figure 3:**
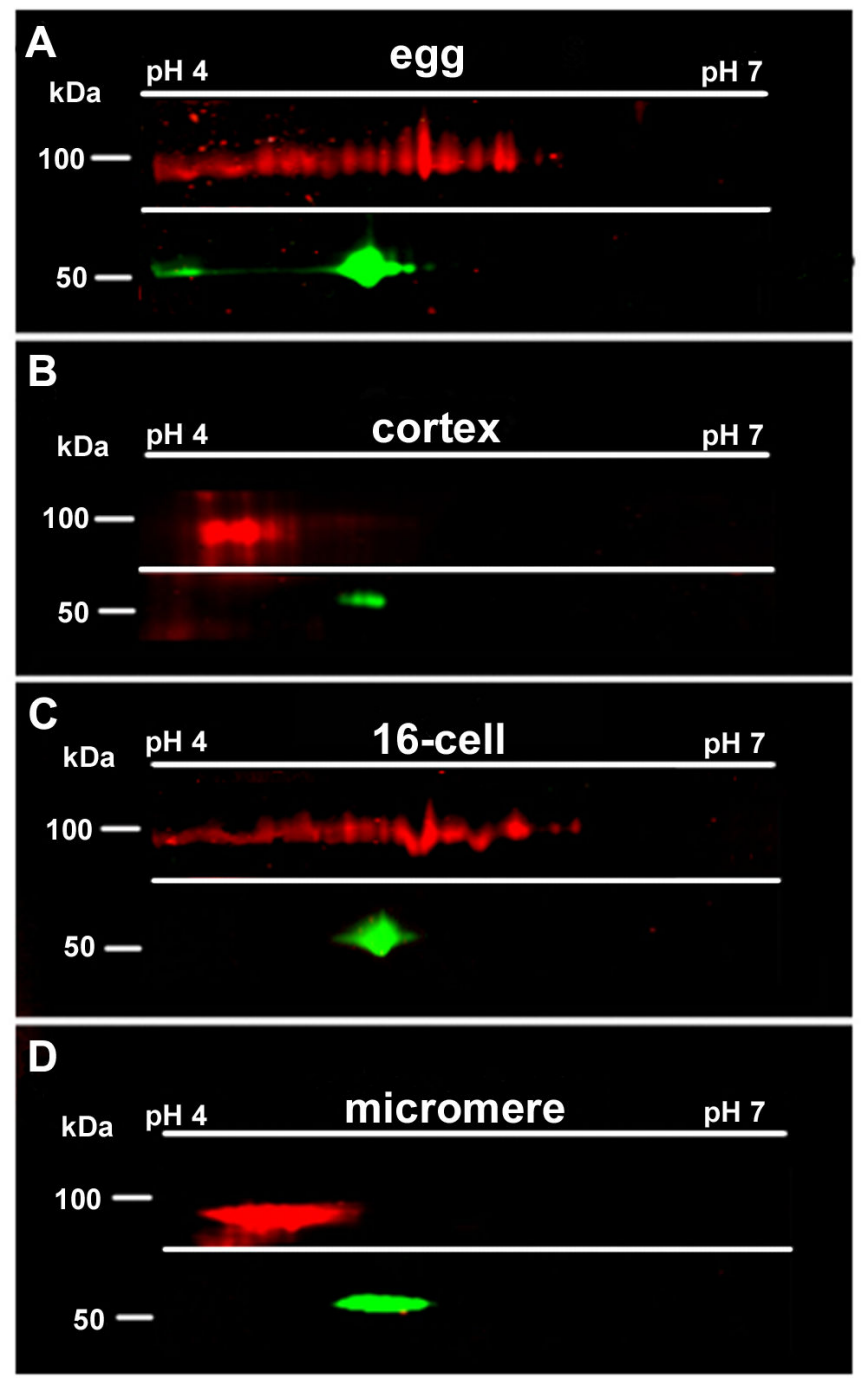
The pool of Disheveled protein in the vegetal cortical domain is differentially post-translationally modified. Approximately 40 mg of total protein from (A) eggs, (B) isolated cortical fragments, (C) 16-cell stage embryos, (D) 16-cell stage micromeres, were collected from *S*. *purpuratus* and then subjected to 2D gel electrophoresis, separating the proteins first by charge (on a pH 4 to pH 7 IPG strip) then by size, and then to Western blot analysis using anti-Dsh antibodies. Dsh (red) isoforms in isolated cortex samples and micromere samples are enriched near the acidic (left) side of the gel, while whole embryo samples show the presence of Dsh protein species that are not detected in the isolated cortex and 16-cell stage micromeres. Tubulin (green) serves as an internal control.

 Several lines of evidence indicate that the “cortical” Dsh is key for the localized activation of the EGRN. First, Dsh protein is broadly expressed in the egg and early embryos, but it is enriched in the VCD. Second, unlike overexpressed β-catenin and dominant-negative GSK-3β which induces ectopic endomesoderm [16,18], overexpressing Dsh in the animal half blastomeres does not produce any perceptible morphological effects on embryo development indicating that Dsh alone is not sufficient for the ectopic activation of the EGRN [31]. Third, removing the vegetal cortex animalizes sea urchin embryos and this effect can be partially reversed by injection of Dsh mRNA [21]. Finally, transplanting and fusing the Dsh-containing VCD to the animal pole of a host egg is sufficient to induce ectopic endoderm at the animal pole [21]. These observations raised the possibility that the VCD-localized Dsh that is subsequently inherited by vegetal blastomeres is differentially regulated to mediate its localization and/or activation in this domain. To investigate this possibility, we carried out 2D Western blot analysis on eggs, isolated egg cortices, 16-cell stage embryos and isolated 16-cell stage micromeres. The results from this analysis showed that there is a strikingly different pattern of Dsh post-translational modification in the isolated cortices and micromeres compared to the pattern seen in whole egg and embryo extracts (Figure 3). Dsh isoforms in whole eggs and 16-cell stage embryos displayed a wide range of isoelectric points (pI) that stretched from a pH range of 4.2 to 6.6 (Figure 3A, C). Strikingly, compared to the 2D Western blot patterns of Dsh from eggs and 16-cell stage embryos, Dsh isoforms in the cortical fragment and micromere extracts looked similar and they were predominantly restricted to the acidic pI range between pH 4 and 5 (Figure 3B, D). We conclude that Dsh protein localized to the VCD is differentially modified by post-translational modification compared to Dsh isoforms in the bulk cytoplasm. At this time we do not know if the post-translational modifications of Dsh in the VCD and in the micromeres are the same. However, since the cWnt pathway is first activated in the 16-cell stage micromeres, our results provide a potential assay to examine selective Dsh regulation in these cells during cWnt activation. 

 The molecular basis for Dsh modification in the VCD is not known but it likely involves phosphorylation since many studies have identified phosphorylated residues on Dsh homologs from various species [42,43]. Our attempts to determine if the modifications seen in VCD-localized Dsh in sea urchin eggs are due to phosphorylation were not successful due to degradation of the protein during the phosphatase treatments even in the presence of a cocktail of protease inhibitors (unpublished observations). However, our results clearly demonstrated that endogenous Dsh is differentially post-translationally modified in different cellular compartments. In sum, the observations reported above provide insight into the initial specification of the AV axis and the mechanisms that selectively regulate activation of cWnt signaling in vegetal blastomeres during endomesoderm specification in the sea urchin embryo.

### Dsh protein accumulation in the VCD during oogenesis indicates that an AV polarity is established in early oocytes

**Figure 4:**
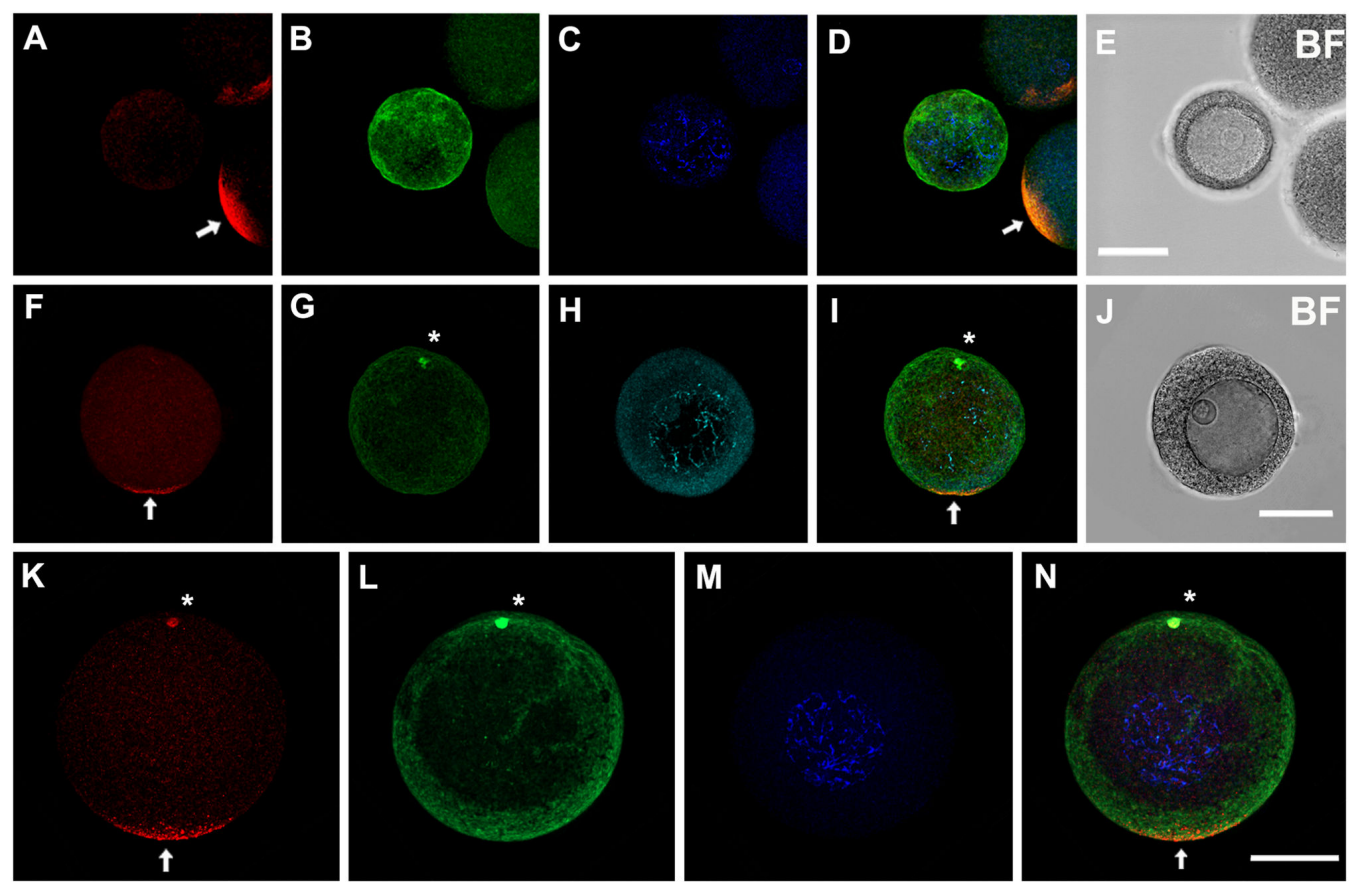
Disheveled accumulation in the vegetal cortical domain begins during early oogenesis. Immunolocalization of Dsh during oogenesis in *Lytechinus* *pictus*. Sections of the ovary were dissected and oocytes at different stages were released by gentle shaking of the tissue in seawater. (A-E) No Dsh protein is detected in primary oocytes. Cortical Dsh localization is detected in the mature egg on the right of the primary oocyte (white arrows in A and D). (E) Bright field view. (F-J) Midsize oocytes showing the first detectable accumulation of Dsh in the vegetal cortical domain (white arrow). Note the MTOC at the opposite end of the Dsh staining (white asterisk). (J) Bright field view. (K-N) Large oocytes show strong Dsh labeling in the vegetal cortical domain (white arrow). Note that strong Dsh staining is also seen in the MTOC (white asterisk) at this stage. Expression of Dsh protein is shown in red, filamentous actin is visualized with fluorescein phalloidin (green) and nuclei are visualized with DAPI staining (blue). Arrows indicate Dsh staining and the asterisks indicate the MTOC. (BF) Bright field view.

 In the sea urchin, it has been well established that the AV axis is specified during oogenesis [8,44]. However, the lack of clear molecular markers for the AV axis in oocytes has made it difficult to determine when this maternal anisotropy is initially established. The VCD clearly plays a key role in specification of the AV axis and the anti-SUDsh antibodies provided useful reagents for identifying when a molecular asymmetry is first established in the sea urchin oocyte. To determine when the Dsh protein begins to accumulate in the VCD during oogenesis we carried out immunolocalization of Dsh in oocytes at different oogenic stages obtained from excised ovaries. We noted that the smallest oocytes showed low levels of Dsh expression throughout the cytoplasm and these oocytes did not have the microtubule organizing center (MTOC) that forms at the animal pole prior to meiosis and polar body formation [45] (Figure 4A-E). We observed that the Dsh protein begins to accumulate asymmetrically in the cortex of small oocytes and this pole was identified as the vegetal pole by the presence of an MTOC at the opposite pole of the cell (Figure 4F-J). MTOC staining was further confirmed by using an anti-γ-tubulin antibody [46] (Figure S3A-F). It is notable that Dsh accumulation at the vegetal cortex is strongly correlated with the appearance of the MTOC, and this observation raises the possibility that enrichment of this protein in the VCD might be linked with cellular events that lead to MTOC formation in the oocyte. In mid-stage oocytes we noted a relatively wide domain of Dsh accumulation at the vegetal cortex concentrated in punctate structures (Figure 4K-N). Interestingly at this stage some of the Dsh puncta appeared to reside in the cytoplasm at the vegetal pole, but not closely associated with the egg cortex (Figure 4K). The presence of these puncta in the cytoplasm suggests that Dsh may be transported to the VCD in a larger protein complex. Alternatively, the puncta may represent polyribosomes translating *Dsh* mRNA, which might indicate that the Dsh protein is synthesized at a higher rate at the vegetal pole. Further studies are needed to distinguish between these possibilities. We conclude that the VCD is formed very early during oogenesis and suggest that accumulation and selective postranslational modification of Dsh on this scaffold marks the vegetal pole for endomesoderm specification later in development. 

 Interestingly, we noted that Dsh staining is also seen in the MTOC at the animal pole in mid-stage oocytes (Figure 4K-N). The function of Dsh in the MTOC in the sea urchin oocyte is not known, but this observation adds to the many studies that have localized Wnt pathway components to the centrosomes and/or basal bodies in various cell types [47-49]. 

### Dsh localization and stability in the egg are affected by disruption of microfilaments

 The accumulation of differentially modified forms of Dsh in the VCD raises several interesting questions about the mechanisms establishing and maintaining this novel domain in sea urchin eggs. One relevant question relates to the molecular mechanisms underlying the anchoring of Dsh to the VCD. Several observations made in the current study and in previous studies raised the possibility that Dsh is anchored on the vegetal cortex by components of the cytoskeleton. First, immunostaining of the egg cortices showed that Dsh puncta are embedded between the actin filaments, which suggested that the Dsh protein is associated with structures that are anchored on the cortex by cortical F-actin (Figure 2L). In addition, Dsh contains known microtubule and microfilament binding domains and it has been shown to interact with these cytoskeletal components [50]. Moreover, extensive arrays of microfilaments, microtubules and cytokeratin-type intermediate filaments and concentrated myosin II have been shown to be present in the cortex of unfertilized eggs, although none of these molecules have been shown to be expressed asymmetrically in the egg cortex [46,51,52]. To determine if specific cytoskeletal elements are involved in anchoring Dsh to the vegetal cortex, unfertilized eggs were treated with cytochalasin and colchicine to selectively disrupt actin filaments and microtubules respectively. 

**Figure 5:**
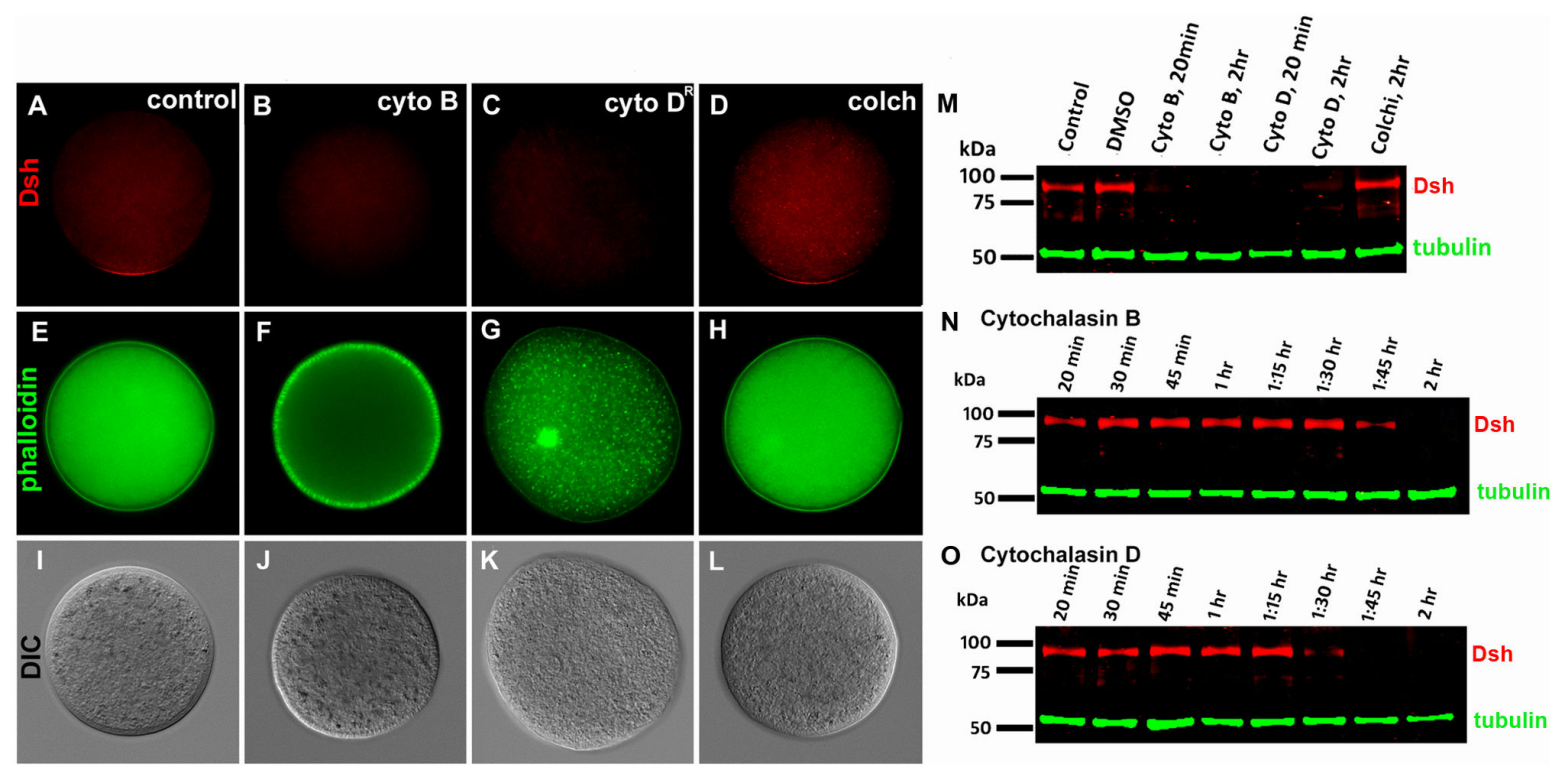
The effect of cytoskeletal disruption on Disheveled localization and stability in the sea urchin egg. (A-L) The effect of disrupting microfilaments and microtubules on the localization of Dsh to the vegetal cortical domain. *S*. *purpuratus* eggs were treated with DMSO (A, E, I), cytochalasin B (B, F, J), cytochalasin D (C, G, K) or colchicine (D, H, L) for 20 minutes, washed and then incubated for a total time period of two hours. Following the incubation the eggs were processed for Dsh immunofluorescence (A-D) and for F-actin staining using fluorescein phalloidin (E-H). (I-L) are bright field views of the corresponding fluorescent images. (A) Control DMSO treated eggs show Dsh localization at one pole of the egg. (B) and (C) Dsh localization was abolished in eggs treated with 10 mg/ml cytochalasin B or D for 20 minutes, washed and incubated in seawater for a total time of 2 hours. (D) Eggs treated with 100 mM colchicine for 2 hours showed no effect on Dsh localization to the vegetal cortical domain. (M-O) Western blot analysis of Dsh protein in *S*. *purpuratus* eggs following cytochalasin and colchicine treatment. (M) The effect of cytochalasin and colchicine treatment on the stability of Dsh in *S*. *purpuratus* eggs. In some cases (lanes 2, 3, 5) the eggs were exposed to DMSO or the cytoskeletal disrupting drugs for 20 minutes, washed and then incubated for a total time of 2 hours prior to processing them for Western blot analysis. In other cases (lanes 4, 6, 7) the eggs were left in the drugs for the 2 hour incubation period prior to processing for Western blot analysis. Untreated control eggs (lane 1) , DMSO- (lane 2), and colchicine-treated (lane 7) eggs express the Dsh protein, while there is no Dsh detected in the cytochalasin-treated eggs (lanes 3-6). (N, O) Eggs were briefly exposed to either cytochalasin B or D for 5 minutes and then incubated in seawater for approximately 2 hours. Samples were collected for Western blot analysis at the times indicated in the figure. Loss of Dsh protein reactivity on the Western blots can be observed starting at 1:45 hr in cytochalasin B treated eggs, and at around 1:30 hours in cytochalasin D treated eggs. The same batch of eggs was used for immunofluorescence and Western blot analyses.

 The cytochalasins B and D are known to disrupt F-actin and induce reorganization of cortical actin filaments into actin patches or short rods [53]. Cytochalasin B is an irreversible disruptor of F-actin and hence we also used the reversible F-actin inhibitor cytochalasin D to determine the effect of transient microfilament disruption on Dsh localization and stability in the unfertilized egg. Morphological changes were induced in the eggs within minutes after addition of the cytochalasins indicating that microfilament disruption took place very rapidly (data not shown) and phalloidin staining confirmed that these drugs disrupt the microfilaments in sea urchin eggs as shown in other studies [54] (Figure 5). Immunolocalization of Dsh in cytochalasin-treated eggs showed that accumulation of the protein in the VCD was unaffected even after 1 or 1.5 hours of incubation in the drugs (not shown). After approximately 2 hours of incubation time control eggs (Figure 5A, E, I) and colchicine treated eggs (Figure 5D, H, L) showed robust Dsh staining in the VCD but we noted the loss of Dsh from the VCD following disruption of microfilaments with cytochalasin B (Figure 5B, F, J) and cytochalasin D (Figure 5C, G, K). We also noted that these eggs also appeared to lose Dsh immunoreactivity in the cytoplasm (compare Figure 5A and 5D with Figures 5B and 5C). This raised the possibility that disruption of microfilaments was leading to the destabilization of all Dsh pools in the egg. To evaluate this possibility we performed Western blot analysis of control and drug treated eggs. In some cases we treated the eggs for 20 min with the cytochalasins and then rinsed them before further incubation for another 100 min. In other experiments we let the eggs incubate in the drugs for the two hour duration of the treatment. Western blot analysis showed that there is a striking downregulation of Dsh protein in eggs treated with the cytochalasins after approximately 2 hours following the addition of the microfilament disruptors (Figure 5M). Dsh stability was not affected by disruption of microtubules with colchicine (Figure 5M). To determine when Dsh protein stability was being affected following the disruption of F-actin, we treated eggs with either cytochalasin C or D for 5 minutes, then washed out the drugs and collected samples for Western blot analysis at different time points. This analysis also confirmed that the Dsh protein appears to be relatively stable for about 1.5 hours after drug treatment before undergoing degradation over a period of 15 to 30 minutes (Figure 5N, O).

 Since disruption of microfilaments did not result in a rapid delocalization of Dsh from VCD puncta, we conclude that microfilaments are not involved in the direct tethering of Dsh to the VCD. However, the global downregulation of Dsh throughout the egg after a brief pulse of cytochalasin D indicated that normal microfilament structure is required for maintenance of VCD integrity and Dsh stability. It is possible that disruption of cortical microfilaments leads to the loss of the scaffold that forms the VCD, but since we do not have another marker for the VCD, we cannot distinguish between loss of the VCD scaffold and loss of Dsh associated with the VCD. However, loss of VCD integrity alone cannot explain the striking downregulation of Dsh in the entire egg after a pulse of cytochalasin D, which should have allowed the eggs to reassemble microfilaments. This result is discussed further below. 

### The female pronucleus does not mark the animal pole in *S. purpuratus* and *Lytechinus pictus* eggs

 Anti-SUDsh antibody staining experiments using unfertilized eggs from *S. purpuratus* and *L. pictus*, showed that the female pronucleus is rarely located opposite the domain of Dsh staining at the vegetal pole. This observation was inconsistent with an earlier study by Di Carlo et al. [55] that reported that in the sea urchin *P. lividus*, the female pronucleus is consistently located at the animal pole in mature eggs. During sample preparation for our immunostaining experiments we did not centrifuge the eggs at any step in the staining procedure. Hence, the apparent random location of the female pronucleus we observed was not likely due to its displacement during centrifugation. To resolve the discrepancy between our results and those of Di Carlo et al. [55] we used another animal pole marker to examine the location of the female pronucleus in live *S. purpuratus* and *L. pictus* eggs. In sea urchins and sand dollars, the animal pole can be identified by the position of the jelly canal and studies have shown that the release of polar bodies corresponds to the location of this structure [44,56]. The jelly canal can be revealed by immersing unfertilized eggs in India ink [56]. To determine the relationship between the female pronucleus and the jelly canal in *S. purpuratus* and *L. pictus* eggs we immersed them in Sumi ink and observed the position of the jelly canal with respect to the pronucleus. Our observations clearly indicated that in these two species of sea urchins the pronucleus is positioned randomly relative to the jelly canal (Figure S4). Only 2% (1 out of 50 eggs counted) and 8% (4 out of 50 eggs counted) of the female pronuclei are located directly below the jelly canal in *S. purpuratus* and *L. pictus* respectively. This result indicates that after oocyte maturation in these species, the female pronucleus moves randomly in the egg and is hence rarely found located opposite the vegetal pole in eggs immunostained with anti-SUDsh antibodies.

## Discussion

 Experimental studies have provided compelling evidence that the vegetal cortex of the sea urchin egg is required for cWnt-mediated activation of the EGRN in vegetal blastomeres, but the underlying mechanisms have been unclear. In the current study we have shown that the VCD, a novel domain in the sea urchin egg cortex, sequesters a differentially modified pool of Dsh protein at the vegetal pole during early embryogenesis. Several lines of evidence indicate that the VCD may serve as a molecular scaffold to locally “activate” Dsh signaling and hence restrict early cWnt signaling to vegetal blastomeres in the sea urchin embryo. This work provides novel insights into how a cytoarchitectural polarity in the egg cortex could be used as a molecular scaffold to locally activate a gene regulatory network mediating germ layer segregation during early embryonic development. 

### Differential regulation of Dsh along the animal-vegetal axis in sea urchins

 In sea urchins *Dsh* mRNA is uniformly distributed in eggs and early embryos with no apparent asymmetric spatial enrichment of the message during these stages. But several lines of evidence indicate that Dsh protein localization and its activity in the cWnt pathway are differentially regulated along the AV axis by distinct mechanisms. In the current study we demonstrated that while endogenous Dsh protein is broadly distributed in the egg and early embryo, there is an accumulation of the protein in a punctate pattern in the VCD. The observation that endogenous Dsh accumulates in the VCD is consistent with previous studies that have shown that a Dsh::GFP fusion protein overexpressed by mRNA injection into fertilized eggs, and endogenous Dsh accumulate at the vegetal pole in a punctate pattern [21,31]. Hence, Dsh overexpressed by mRNA injection seems to be targeted to the VCD. However, in these Dsh::GFP overexpression experiments only very low levels of fluorescence were seen in the cytoplasm [31,32]. Since the mRNA coding for the Dsh::GFP fusion protein is injected into zygotes and it is likely incorporated into all blastomeres, the low levels of fluorescence outside the VCD in Dsh::GFP mRNA-injected zygotes and embryos suggests that the fusion protein is unstable when not localized to the VCD. The degradation of the Dsh protein after disruption of microfilaments suggests that the actin cytoskeleton may play a role in regulating the stability of this protein in the egg. The Dsh protein has a defined actin-binding site and it is known to interact with actin [50,57], and while it is logical to invoke a transport or tethering function for the cytoskeleton in regulating this protein, the degradation of all Dsh pools following cytochalasin treatment is puzzling. This effect does not reflect a general effect on all egg proteins since the disruption of microfilaments does not lead to the degradation of tubulin (Figure 5M-O) or actin (not shown). Moreover, pulse-treating eggs with cytochalasin D, which has a reversible effect on the actin cytoskeleton, for just five minutes (Figure 4O) also led to Dsh degradation in eggs following the two hour incubation period. Further studies are needed to determine how disruption of the cytoskeleton affects the stability of Dsh in the sea urchin egg, and to determine if the stability of other regulatory proteins in the cWnt pathway is affected by microfilament disruption. 

### The potential role of the VCD in selectively activating Dsh-mediated cWnt signaling in vegetal blastomeres

 The recent sequencing of the *S. purpuratus* genome showed that there are eleven Wnt ligands, four Frizzled receptors and one LRP 5/6 receptor in this species [28,58]. Subsequent studies have shown that none of the mRNAs coding for the maternally expressed ligands and receptors is enriched at the vegetal pole of the egg or early embryo [21,22,30]. While it is possible that there are unidentified transmembrane cell surface components in the cWnt pathway that are localized to the vegetal pole, there is now compelling evidence that the determinants that selectively activate cWnt signaling intracellularly in vegetal blastomeres during endomesoderm specification are firmly attached to the VCD. Early insights into the cortical localization of regulatory factors in echinoderm ova came from the work of experimentalists who showed that the removal of vegetal deep cytoplasm had little effect on gastrulation in sea urchins [59]. In sharp contrast, sea urchin and sea star embryos developing from eggs where the vegetal cortex is selectively extirpated [VC (-)] become severely animalized and do not gastrulate [21,60]. Analysis of multiple molecular marker expression in *P. lividus* embryos developing from VC (-) eggs showed that they do not form endoderm or mesoderm [21]. Croce et al. [21] went on to show that VC (-) embryos did not activate the cWnt pathway in vegetal blastomeres and moreover, showed that the animalized phenotype could be rescued by overexpression of activated β-catenin. Overexpression of other Wnt pathway components such as Wnt6, a dominant-negative form of GSK (dnGSK) and overexpression of Dsh could also rescue endomesoderm in these embryos, but to a lesser extent. Unlike VC (-) embryos rescued with activated β-catenin, however, which had a relatively normal larval morphology, the VC (-) embryos where endomesoderm was rescued by overexpression of Wnt6, dnGSK and wild-type Dsh had severely disrupted larval morphology. Additionally, these experiments did not determine if the rescue in endomesoderm was in vegetal cells or in cells derived from the animal half [21]. Overall, these results further support the hypothesis that the vegetal cortex localizes determinants required to activate the cWnt pathway in vegetal blastomeres.

 The results from the current study and our previous observations have provided key insight into how the VCD may play a role in the selective activation of cWnt in vegetal blastomeres by the differential regulation of Dsh. We have previously shown that blocking Dsh function in the cWnt pathway by overexpressing a dominant-negative form of the protein downregulates nuclear β-catenin and animalizes embryos [31]. However, while overexpression of β-catenin vegetalizes sea urchin embryos by respecifying mesomeres as endomesoderm, overexpression of full-length Dsh has no discernible effects on embryos [18,31]. Dsh is a phosphoprotein and previous work in other metazoans has shown that it is post-translationally modified by several kinases [42,43]. In our studies 2D Western blot analysis clearly showed that sea urchin Dsh is present in many isoforms in eggs and early embryos. As previously noted, the VCD contains a differentially modified pool of Dsh isoforms that may be key to the localized activation of cWnt signaling in vegetal blastomeres. The mechanisms that mediate the localization and the differential modification of Dsh in VCD puncta are not known but these two processes are likely to be tightly linked. It is possible that there is a factor, putatively a protein kinase, localized to the VCD that modifies Dsh protein at the vegetal pole. This modification of Dsh may then serve to localize or tether Dsh to the VCD puncta. Alternatively, there may be a more broadly distributed factor that post-translationally modifies Dsh and this change in turn targets Dsh to puncta in the VCD [31]. In this case, the VCD serves as a scaffold for modified Dsh. Further studies are needed to distinguish between these possibilities.

 Our observation that a differentially modified pool of Dsh accumulates in the VCD suggests that this population of Dsh plays a role in activation of cWnt signaling in vegetal blastomeres. However, it is unlikely that the VCD pool of Dsh in the egg represents a form of the protein that is competent to directly activate cWnt signaling since activation of cWnt signaling does not occur until the 16-cell stage when nuclear β-catenin is detected in the micromeres and these cells begin to express target genes of the cWnt pathway [17,23]. It is possible that the pool of Dsh in the egg that is localized to the VCD is a form of the protein that is primed for its activation in the cWnt pathway. This idea is supported by recent work in cultured mammalian cells that has shown that activation of Dsh involves two critical post-translational modification events that have to occur sequentially in cells before Dsh can activate cWnt signaling. In that work Bernatik et al. [61] showed that in cultured mammalian cells Dsh is first phosphorylated by Protein Kinase CK2 (formerly known as CK2) and PAR1, before it can be phosphorylated by Casein Kinase 1. We suggest that the post-translational modification of Dsh in the VCD primes this pool of Dsh for “activation” at the 16-cell stage by a factor that is localized to the vegetal pole. It is striking that the punctate Dsh pools inherited from the VCD are only found in those cells that nuclearize β-catenin at the 60-cell stage of development (Figure 1F). The absence of the VCD may explain why Dsh is unable to ectopically activate cWnt signaling in animal half blastomeres, but further work is needed to investigate the relationships between the VCD puncta, Dsh post-translational modification, and activation of the cWnt pathway in vegetal blastomeres. 

### The initial establishment of polarity in sea urchin oocytes

 Boveri [8,9] was among the first to demonstrate that sea urchin eggs are polarized along the AV axis, and this polarity is manifested as distinct morphological markers in echinoderms. Holothurian (sea cucumbers) oocytes display the most dramatic morphological polarization along the AV axis and in these animals the animal pole can be identified based on the apical protuberance, an apically displaced nucleus [62-64], an apical centriole [63], or a flagellum [62]. Similarly, asteroid (sea star) oocytes express an AV polarity that can be identified by apical centrioles [65,66], an apically displaced nucleus, an absence of large vacuoles, and actin-filled spikes at the animal pole [66]. In several species of echinoids (sea urchins and sand dollars), the animal pole of oocytes can be traced by the location of the jelly canal, the site of polar body extrusion [44,56], and a cortical microtubule-organizing center (MTOC) [45]. Hence, the AV polarity becomes morphologically evident during oogenesis, but exactly when during this process the two poles first become polarized at the molecular level has heretofore been unclear [67]. In this study, we showed that Dsh protein localization is first detected in small oocytes, which suggests that the AV polarity in the sea urchin is established very early during oogenesis. What is not clear however, is the origin of the asymmetry that becomes the VCD in the mature egg. A concentrated accumulation of Dsh in a punctate pattern marks this domain, but it is likely that the VCD is established before Dsh begins to accumulate in this region. It is known that during oogenesis in sea urchins and other echinoderms the future vegetal pole of the oocyte is attached to the basement lamina of the ovarian epithelium [1,67]. We speculate that the VCD is established by the asymmetry that is initially generated as a result of the interaction between the early oocyte and the basement lamina that perdures after egg maturation is complete. It has been shown that the AV axis polarity of oocytes from many species corresponds to the apical basal polarity of the surrounding germinal epithelium [1,2,10,67,68], and hence it is possible that a VCD like asymmetry exists in the vegetal cortex of other animal eggs.

### The evolution of pattern formation along the AV axis

 Work in a number of bilaterian taxa such as ascidians, hemichordates, nemerteans, mollusks, and echinoderms has shown that cWnt signaling is critical for endoderm/endomesoderm formation [17,18,69-74]. Recent studies have also established that β-catenin specifies endoderm in the Cnidaria, the closest outgroup to the bilaterians, indicating an ancient co-option of this pathway for endoderm specification during metazoan evolution over 700 million years ago [75-78]. Intriguingly, in cnidarians and ctenophores, two early emerging phyla that form outgroups to the bilaterians, endoderm specification occurs in animal half blastomeres in contrast to the vegetal pole derived endoderm in bilaterians [79]. The molecular basis for endoderm specification in ctenophores in not known, but the origin of endoderm from animal pole derived blastomeres of diploblasts has led to the idea that endoderm specification and gastrulation evolved at the animal pole [5,75,76,80]. Moreover, it has been proposed that the mechanisms that activate endoderm specification were moved to the vegetal pole after the emergence of the bilaterian last common ancestor [5,76,80]. In the cnidarian *Nematostella vectensis* Dsh protein is localized at the animal pole and mediates endoderm specification at that pole [75,76]. In sea urchins Dsh accumulates at the VCD where it mediates selective activation of endoderm specification in vegetal blastomeres [31] (this study). Dsh is a scaffolding protein that is known to bind to over fifty partner proteins in various species [42,43]. Hence, a hypothetical mechanism to shift the site of endoderm specification from the animal pole to the vegetal pole may have been the relocalization of a key “activator” of Dsh from the animal pole in pre-bilaterians to the vegetal pole of the urbilaterian. The ability to isolate biochemically significant amounts of egg cortices and micromeres from sea urchins now allows us to begin to identify these putative Dsh regulatory factors, and these molecules may provide the insights needed to reconstruct the evolution of pattern formation along the AV axis during metazoan evolution.

## Materials and Methods

### Animal handling and embryo manipulations

 Adult sea urchins, *S. purpuratus* and *L. pictus* were obtained from Marinus, Garden Grove, CA, or from Point Loma Marine Invertebrate Labs, Lakeside, CA and maintained in seawater aquaria at 15°C. Spawning was induced by intracoelomic injection of 0.5M KCl. Embryos were cultured at 15°C in filtered artificial seawater (ASW) in temperature-controlled incubators. Fertilization envelopes were removed prior to analysis by fertilizing eggs in ASW containing 0.25 µM 3-amino-1, 2, 4-triazole (ATA) (Sigma) before passing the embryos through a cell strainer (BD Falcon). For some experiments eggs were dejellied using acidic seawater as described [34].

### In situ hybridization

 In situ hybridization using digoxigenin-labeled probes was performed as previously described [35]. Fixation was carried out overnight at 4°C in a mixture of 4% (w/v) paraformaldehyde (PFA)(Electron Microscopic Sciences, PA), 32.5% (v/v) filtered ASW, 32.5 mM MOPS pH 7.0, 162.5 mM NaCl. The probes were used at a final concentration of 0.1 ng/µL.

### Preparation of anti-Dsh polyclonal antibodies

 The *S. purpuratus* Dsh (SpDsh) protein is 723 amino acids in length and has a predicted molecular weight of 81 kDa. To investigate the spatiotemporal expression pattern of Dsh three affinity-purified anti-Dsh polyclonal rabbit antibodies were generated against three distinct epitopes on the sea urchin Dsh protein. A His-tagged fusion protein of amino acids 1-101 of the *S. purpuratus* Dsh protein (subcloned and expressed using the pET vector, EMD Millipore) that included the DIX domain was used as one antigen. Polyclonal antibodies were also generated using synthetic peptides corresponding to epitopes at the N-terminus (NH_2_-CASVTTDTRGDSQLPPERTG-COOH) and C-terminus (NH_2_-CMVPMMPRQLGSVPEDLSGS-COOH) of Dsh as antigens. Polyclonal antibodies were generated in rabbits and affinity-purified using the immunizing antigens by Bethyl Labs (Montgomery, TX). As one test of specificity of the antibodies, a preadsorption assay was performed by incubating the Dsh antibodies with the peptides (synthesized by Bethyl labs) or with the Dsh-DIX fusion protein at 10-fold molar excess for one hour at room temperature prior to incubating with the samples either for immunostaining or Western blot analysis. Since all three antibodies have been successfully used against all sea urchin species tested thus far (see results) we refer to the antibodies generated against the DIX domain, the N-terminal epitope and the C-terminal epitope as anti-SUDshDIX, anti-SUDsh-N, and anti-SUDsh-C respectively.

### Immunostaining and image analysis

 Embryos, eggs and oocytes were fixed with 4% PFA in phosphate buffered saline (PBS) pH 7.4 for 20 minutes, post fixed with 100% ice cold acetone for 10 minutes and processed for indirect immunofluorescence. Samples were incubated with primary antibodies for an hour at room temperature using the following dilutions: rabbit anti-SUDshDIX antibody (1:400), rabbit anti-SUDsh-C antibody (1:400), rabbit anti-SUDsh-N antibody (1:200), mouse anti-γ-tubulin antibody (1:50, Abcam, ab11316-100). Following this incubation the samples were rinsed and incubated in secondary antibodies conjugated to the AlexaFluor series (Invitrogen) for 45 minutes at room temperature. DAPI (1:1000, Invitrogen) and fluorescein phalloidin (3:100, Invitrogen) were added at the end of 45 minutes and incubated for another 15 minutes before rinsing. Stained eggs and embryos were observed using a Zeiss Axiovert 200 inverted microscope or a Leica SP5 scanning confocal microscope. Captured images were analyzed with ImageJ (NIH) and Volocity (Perkin Elmer Inc.) and the figures were prepared using Adobe Photoshop (Adobe Systems Inc.).

### Staining the jelly canal

The sea urchin egg jelly canal was stained using a procedure adapted from Maruyama et al. [36]. Spawned eggs were collected “dry” by placing the female urchin with the aboral surface facing down on a Petri dish. These eggs were then transferred directly into a small amount of Sumi ink solution (Fueki Nori Company, Japan). Following incubation in the Sumi ink solution for 3-5 minutes the eggs were placed in seawater and observed under a Zeiss Discovery V8 dissecting microscope.

### Isolation of egg cortices and blastomeres

 Cortex fragments were isolated from eggs and zygotes following the procedure of Vacquier [37]. Briefly, dejellied unfertilized eggs or zygotes were attached to poly-lysine-coated slides and then subjected to a stream of cortical lawn isolation buffer (0.8 M mannitol, 50 mM HEPES, 50 mM PIPES, 5 mM EGTA, 2.5 mM MgCl_2_ˑ6H_2_O, pH 6.5) squirted from a rinse bottle. This procedure left large circular cortical fragments that were relatively devoid of cytoplasmic material attached to the glass. The samples for immunostaining were fixed with 4% PFA, immunostained and observed using scanning confocal microscopy as described above. The cortex samples for Western blot analysis were collected on poly-lysine-coated 60x15 mm Petri dishes using the procedure described above. Cortical fragments were lysed by adding solubilizing solution (40 mM Tris base, 2% SDS, 100 mM DTT) containing a protease inhibitor cocktail (Roche Applied Science, Indianapolis) directly to the dishes. Sample preparation for SDS-PAGE was done as described below.

 Purified micromeres were collected following the procedure described by Wilt and Benson [38] with modifications of the linear sucrose gradient. Embryos at the 16-cell stage were completely dissociated and the cells were layered on top of a discontinuous sucrose gradient of 4% (w/v) and 16% (w/v) in calcium-free seawater. The different sized blastomeres were then separated by sedimentation at 1 x g for 1 hr on ice. The micromeres were retained in the 4 % sucrose while the larger blastomeres sedimented between the interface of 4% and 16% sucrose. Micromeres in the 4% sucrose were collected and centrifuged for one minute at 86 x g to remove the 4% sucrose before addition of the solubilizing solution for two dimensional (2D) SDS-PAGE. 

 Mesomere and macromere/micromere pairs were collected from dissociated embryos using the procedure described in Wikramanayake et al. [39]. These samples were processed for SDS-PAGE and Western blot analysis using the same procedure as described for other samples in this study.

### Western blot analysis

 Egg and embryo samples were collected and centrifuged for one minute at 86 x g. After removing as much seawater as possible, the embryos were lysed in 100 µl of solubilizing solution (40 mM Tris base, 2% SDS, 100 mM DTT) and protein concentrations were determined using the Bradford assay (Bio-Rad). Samples were then mixed with Laemmli sample buffer [40] and boiled for 5 minutes. A total of 30 µg of protein from each sample was run on a 10% SDS-PAGE gel and transferred onto Trans-Blot nitrocellulose membranes (Bio-Rad). Immunoblots were probed with rabbit anti-SUDsh-DIX antibody (1:1000) and mouse anti-tubulin (1:1000) (Developmental Studies Hybridoma Bank, E7). Blots were developed by chemiluminescence (Thermo Scientific, Rockford, IL) or detected with IRDye 680 (1:15,000) and IRDye 800 (1:15,000) secondary antibodies (LI-COR, Lincoln, NE) respectively using a LI-COR Odyssey Infrared Imaging System. 

 2D Western blot analysis of unfertilized eggs, 16-cell stage embryos, and micromeres was carried out using the following procedure. Samples were collected in a 1.5 ml Eppendorf tube and centrifuged at 86 x g for one minute to collect a tight pellet. The seawater was then decanted using a pipette to remove as much seawater as possible. Isolated egg cortices were collected as described above. Pelleted samples were lysed in 100 µl of solubilizing solution. The protein concentrations were determined using the Bradford assay (Quick Start Bradford Dye Reagent, BIO-RAD) and then the samples were desalted using the ReadyPrep 2D cleanup kit (BIO-RAD). For 2D SDS-PAGE 40 µg of total protein of each sample was solubilized in 125 µl of 2D electrophoresis buffer (ReadyPrep 2D Rehydration/Sample Buffer 1; 7 M urea, 2 M thiourea, 1% (w/v) ASB-14, 40 mM Tris, 0.001% Bromophenol Blue, BIO-RAD) with the addition of 25 mM DTT, 4% (w/v) CHAPS, and 0.2% Bio-Lyte pH 3-10/4-7 (2:1)(BIO-RAD). Samples were loaded on an immobilized pH gradient (IPG) ReadyStrips 4-7 (BIO-RAD) and isoelectrofocused with the Protean IEF cell (BIO-RAD) by applying a total of 10,000 V-hr according to the ReadyStrip IPG Instruction Manual (BIO-RAD). The IPG strips were then equilibrated in SDS-PAGE equilibration buffer (6 M urea, 0.375 M Tris-HCl, pH 8.8, 2% SDS, 20% glycerol, 2% (w/v) DTT) two times (20 minutes each) and transferred to a 10% Tris-Glycine Polyacrylamide Gel. SDS-PAGE was performed and the proteins were transferred onto Trans-Blot nitrocellulose membranes (Bio-Rad). Immunoblots were probed with the anti-SUDshDIX and anti-tubulin antibodies as described earlier. 

### Cytoskeleton disruption studies

 To determine if specific components of the cytoskeleton are involved in localizing or tethering Dsh to the vegetal cortex of the egg, specific cytoskeletal components were disrupted using the following chemicals: cytochalasin B (10 µg/ml), cytochalasin D (10 µg/ml), and colchicine (100 µM) (all inhibitors were obtained from Sigma Aldrich). All the inhibitor stock solutions used dimethyl sulfoxide (DMSO) as the solution vehicle, and controls included DMSO at the same concentrations expected in the diluted inhibitor solutions used in the assays. Eggs were dejellied prior to applying the respective inhibitors. Treated eggs were incubated at 15°C and samples were collected for immunostaining and Western blot analysis after 2 hours following drug treatment. Recovery experiments were done by exposing eggs to the drugs for 20 minutes, washing the eggs three times with FSW, followed by incubation of the eggs for an additional 100 minutes at 15°C. To determine the timing of Dsh degradation following disruption of microfilaments, unfertilized eggs were treated with cytochalasin B or D for 5 minutes, rinsed three times in ASW, and samples were collected for immunostaining and Western blot analysis at different time points.

## Supporting Information

Figure S1 **Disheveled is broadly expressed throughout embryogenesis in sea urchin eggs and embryos.** (A) In situ hybridization detection of *Dsh* mRNA in early stage embryos shows that *Dsh* is ubiquitously expressed starting in the unfertilized egg to the 60-cell stage embryo. (B) Dsh protein is expressed at different developmental stages. Tubulin serves as the loading control. (C) Western blot analysis of isolated animal and vegetal halves from 16-cell stage embryos shows Dsh is expressed in both halves. Actin serves as the loading control. All the samples used in these experiments were collected from *S. purpuratus.*
(TIF)

Figure S2 **Preadsorption assays support the specificity of anti-Dsh antibodies.** (A) SpDsh Western blot. When the affinity-purified anti-SUDshDIX polyclonal antibodies were preadsorbed with a tenfold molar excess of the SpDshDIX fusion protein the SpDsh band was eliminated from the Western blot. Preadsorption of the affinity-purified SUDshDIX antibodies with a ten-fold molar excess of either the Dsh N or the Dsh C peptides did not affect the binding of the SUDshDIX antibodies to the SpDsh protein on the Western blot. (B) Dsh Immunostaining. When the affinity-purified SUDshN or SUDshC antibodies were preadsorbed with a ten-fold molecular excess of the Dsh N or the Dsh C peptides used for generating the respective antibodies, the staining pattern at the vegetal cortex of *S. purpuratus* eggs or 32-cell stage embryos was eliminated. The non-preadsorbed staining pattern is shown in the top two (top: fluorescence images; bottom: corresponding bright field views), and the staining pattern with the preadsorbed antibodies is shown in the two bottom panels.
(TIF)

Figure S3 **The vegetal cortical Disheveled domain is positioned directly across from the microtubule-organizing center.** Oocytes were collected from *S. purpuratus* ovaries, processed for immunofluorescence using anti-Dsh and anti-γ-tubulin antibodies, and viewed using fluorescence microscopy. F-actin was detected using fluorescein phalloidin. (A-C) Mid-stage oocyte double labeled with anti-Dsh antibodies (A) and γ-tubulin antibodies (B). (C) Merged view showing that Dsh protein is localized across from the MTOC. (D-F) Midstage oocyte double labeled with fluorescein phalloidin (D) γ-tubulin antibodies (E) confirming that the F-actin enriched structure is the MTOC. (F) Merged view of (D) and (F).
(TIF)

Figure S4 **The female pronucleus is not localized at the animal pole in *S*.** ***purpuratus* and *L. pictus* eggs.**
Unfertilized eggs were collected and immersed directly into Sumi ink to visualize the jelly canal in (A) *S. purpuratus* and (B) *L. pictus* eggs to examine if the female pronucleus (white asterisk) is at the animal pole as indicated by the stained jelly canal (black asterisk). Only 2 % (1 out of 50 eggs counted) and 8% (4 out of 50 eggs counted) of the female pronuclei are located directly below the jelly canal in *S. purpuratus* and *L. pictus* respectively.
(TIF)
