# Supplementary figures and images for: Differential Regulation of Disheveled in a Novel Vegetal Cortical Domain in Sea Urchin Eggs and Embryos: Implications for the Localized Activation of Canonical Wnt Signaling
Source: PLoS One. 2013 Nov 13;8(11):e80693. doi: 10.1371/journal.pone.0080693 (PMC3827468; doi:10.1371/journal.pone.0080693)

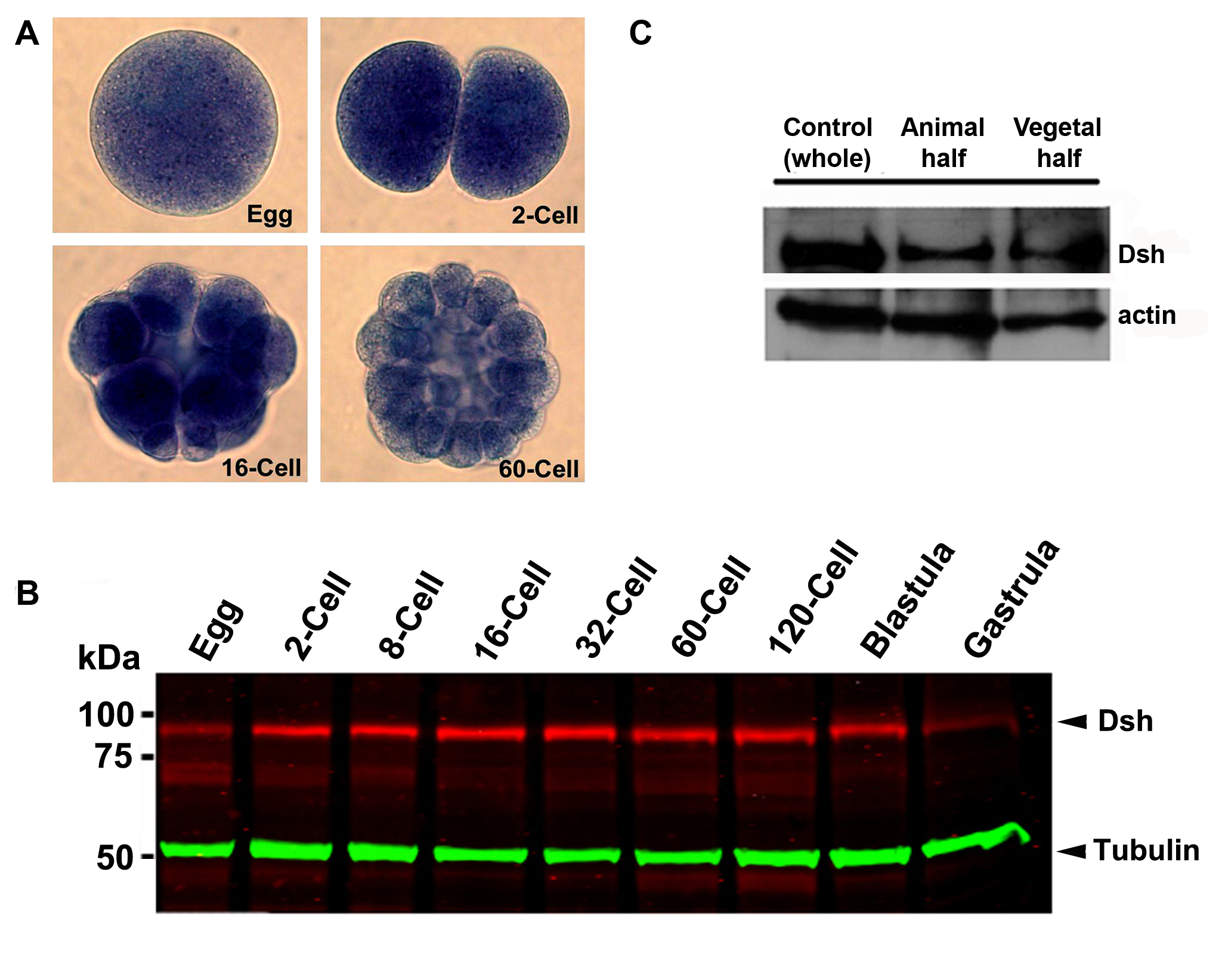

Supplement: Figure S1 — Disheveled is broadly expressed throughout embryogenesis in sea urchin eggs and embryos. (A) In situ hybridization detection of Dsh mRNA in early stage embryos shows that Dsh is ubiquitously expressed starting in the unfertilized egg to the 60-cell stage embryo. (B) Dsh protein is expressed at different developmental stages. Tubulin serves as the loading control. (C) Western blot analysis of isolated animal and vegetal halves from 16-cell stage embryos shows Dsh is expressed in both halves. Actin serves as the loading control. All the samples used in these experiments were collected from S. purpuratus. (TIF) [file pone.0080693.s001.tif]

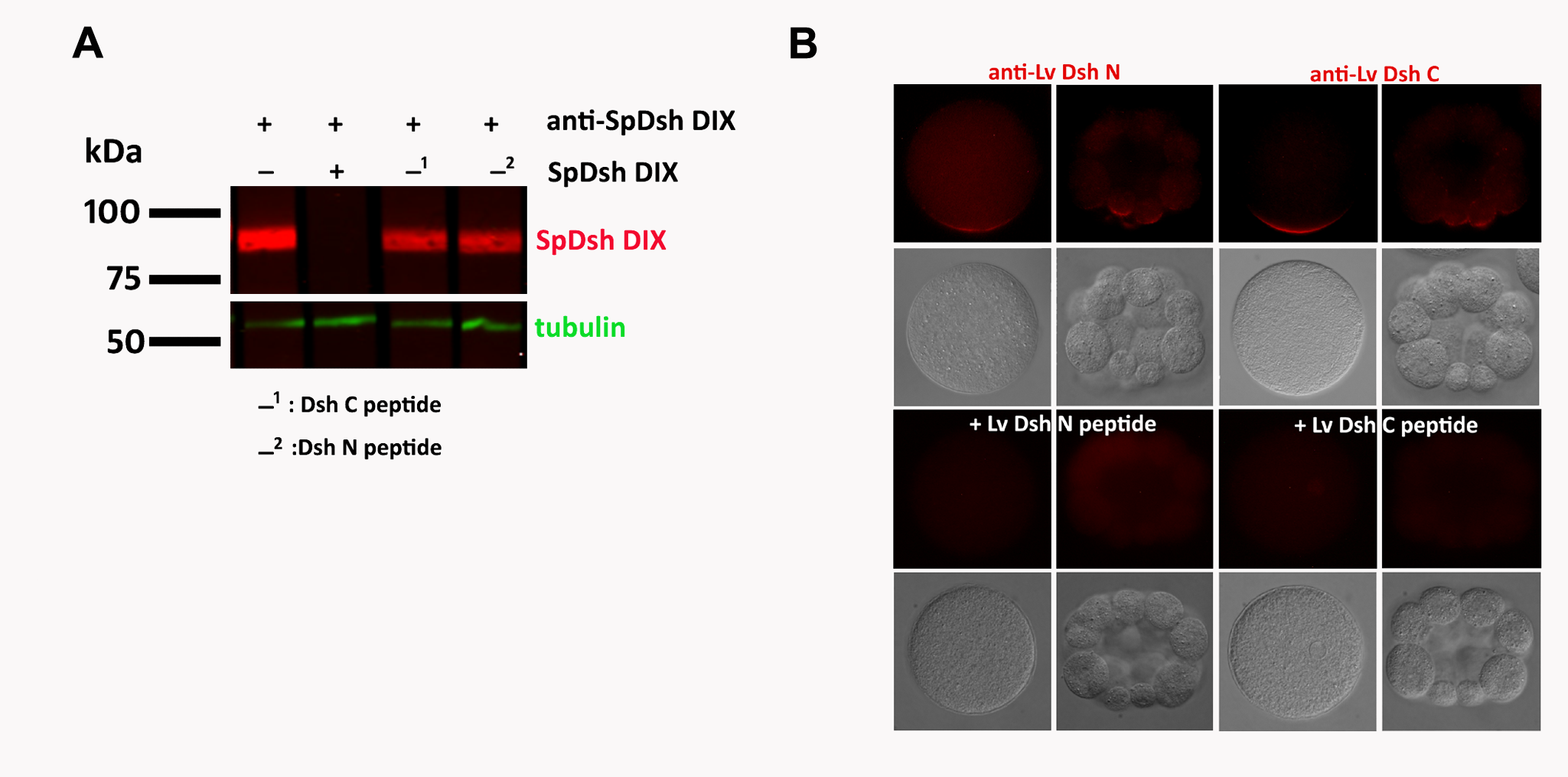

Supplement: Figure S2 — Preadsorption assays support the specificity of anti-Dsh antibodies. (A) SpDsh Western blot. When the affinity-purified anti-SUDshDIX polyclonal antibodies were preadsorbed with a tenfold molar excess of the SpDshDIX fusion protein the SpDsh band was eliminated from the Western blot. Preadsorption of the affinity-purified SUDshDIX antibodies with a ten-fold molar excess of either the Dsh N or the Dsh C peptides did not affect the binding of the SUDshDIX antibodies to the SpDsh protein on the Western blot. (B) Dsh Immunostaining. When the affinity-purified SUDshN or SUDshC antibodies were preadsorbed with a ten-fold molecular excess of the Dsh N or the Dsh C peptides used for generating the respective antibodies, the staining pattern at the vegetal cortex of S. purpuratus eggs or 32-cell stage embryos was eliminated. The non-preadsorbed staining pattern is shown in the top two (top: fluorescence images; bottom: corresponding bright field views), and the staining pattern with the preadsorbed antibodies is shown in the two bottom panels. (TIF) [file pone.0080693.s002.tif]

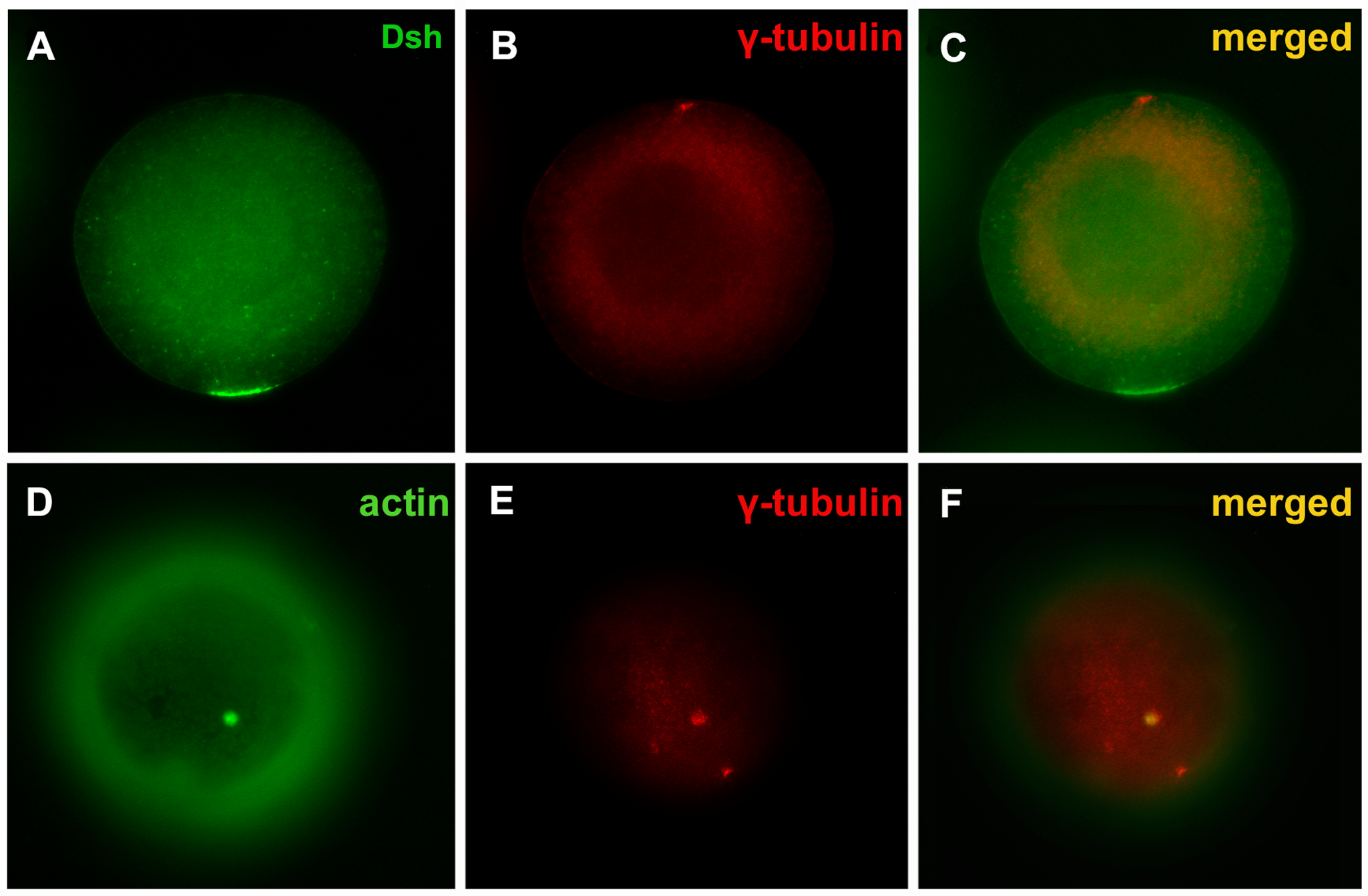

Supplement: Figure S3 — The vegetal cortical Disheveled domain is positioned directly across from the microtubule-organizing center. Oocytes were collected from S. purpuratus ovaries, processed for immunofluorescence using anti-Dsh and anti-γ-tubulin antibodies, and viewed using fluorescence microscopy. F-actin was detected using fluorescein phalloidin. (A-C) Mid-stage oocyte double labeled with anti-Dsh antibodies (A) and γ-tubulin antibodies (B). (C) Merged view showing that Dsh protein is localized across from the MTOC. (D-F) Midstage oocyte double labeled with fluorescein phalloidin (D) γ-tubulin antibodies (E) confirming that the F-actin enriched structure is the MTOC. (F) Merged view of (D) and (F). (TIF) [file pone.0080693.s003.tif]

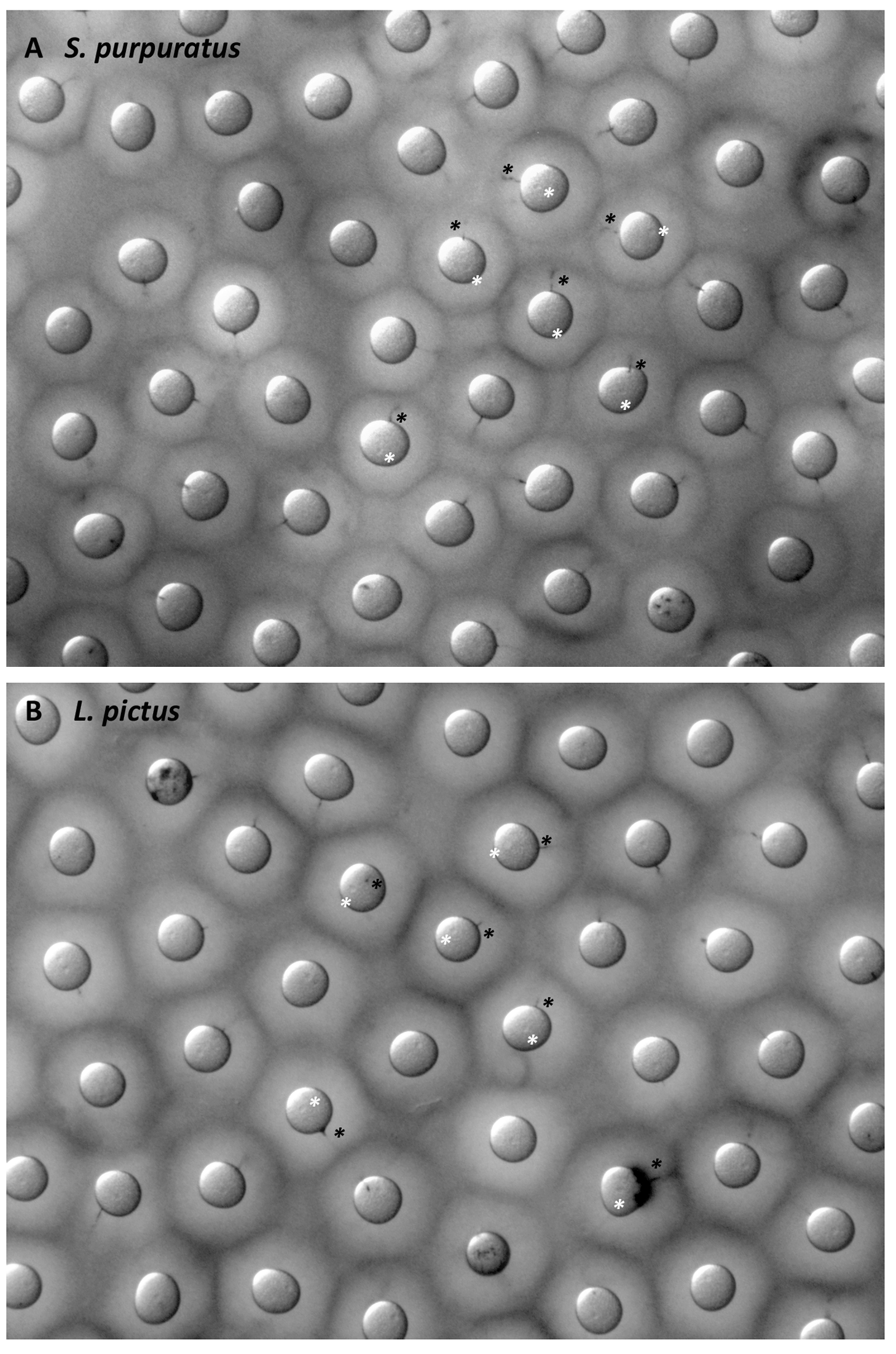

Supplement: Figure S4 — The female pronucleus is not localized at the animal pole in S. purpuratus and L. pictus eggs. Unfertilized eggs were collected and immersed directly into Sumi ink to visualize the jelly canal in (A) S. purpuratus and (B) L. pictus eggs to examine if the female pronucleus (white asterisk) is at the animal pole as indicated by the stained jelly canal (black asterisk). Only 2 % (1 out of 50 eggs counted) and 8% (4 out of 50 eggs counted) of the female pronuclei are located directly below the jelly canal in S. purpuratus and L. pictus respectively. (TIF) [file pone.0080693.s004.tif]
